# Testing the association between social capital and health over time: a family-based design

**DOI:** 10.1186/1471-2458-13-665

**Published:** 2013-07-17

**Authors:** Giuseppe N Giordano, Juan Merlo, Henrik Ohlsson, Maria Rosvall, Martin Lindström

**Affiliations:** 1 Center for Primary Health Care Research, Lund University/Region Skåne, Malmö, Sweden; 2 Unit for Social Epidemiology, Department of Clinical Sciences, Faculty of Medicine, Lund University, Malmö, Sweden; 3 Unit for Social Medicine and Health Policy, Department of Clinical Sciences, Faculty of Medicine, Lund University, Malmö, Sweden; 4 Department of Public Health and Environment, Region Skåne, Malmö, Sweden; 5 Centre for Economic Demography, Lund University, Lund, Sweden

**Keywords:** Self-rated health, Social capital, Trust, Multilevel, Longitudinal, Family-based design, United Kingdom

## Abstract

**Background:**

The past decade has seen a vast increase in empirical research investigating associations between social capital and health outcomes. Literature reviews reveal ‘generalized trust’ and ‘social participation’ to be the most robust of the commonly used social capital proxies, both showing positive association with health outcomes. However, this association could be confounded by unmeasured factors, such as the shared environment. Currently, there is a distinct lack of social capital research that takes into account such residual confounding.

**Methods:**

Using data from the United Kingdom’s British Household Panel Survey (BHPS) (waves thirteen to eighteen, N = 6982), this longitudinal, multilevel study investigates the validity of the association between trust, social participation and self-rated health using a family-based design. As the BHPS samples on entire households, we employed ‘*mean*’ and ‘*difference from the mean*’ aggregate measures of social capital, the latter of which is considered a social capital measurement that is not biased by the shared environment of the household. We employed Generalized Estimating Equations for all analyses, our two-level model controlling for correlation at the household level.

**Results:**

Results show that after adjusting for the shared environment of the household over a six year period, the association between social participation and self-rated health was fully attenuated (OR = 0.97 (95% confidence interval 0.89-1.06)), while the association with trust remained significant (OR = 1.11 (1.02-1.20)). Other health determinants, such as being a smoker, having no formal qualifications and being unemployed maintain their associations with poor self-rated health.

**Conclusions:**

The association between social capital (specifically trust and social participation) and self-rated health appear to be confounded by shared environmental factors not previously considered by researchers. However, the association with trust remains, adding to existing empirical evidence that generalized trust may be an independent predictor of health.

## Background

For well over a century, empirical research has demonstrated a societal influence on individual health outcomes [1]. Since its introduction into the public health arena fifteen years ago [2], social capital research has attempted to offer new insight into possible mechanisms behind societal influences on health (for examples see: [3-5]). However, an on-going lack of consensus regarding the definition and conceptualization of social capital has created disparity amongst researchers, resulting in fervent critique of this field of research [6-8].

Despite the lack of a single, universally accepted social capital theory, a large body of empirical social capital research within the public health arena has adopted Robert Putnam’s theories and definition above all others [9]. One possible explanation is that Putnam’s ‘macro’ view of social capital is more appealing when attempting to operationalize social capital in public health research (compared with the more ‘micro’-oriented view of most sociologists, as described by [10]).

Putnam defined social capital as ‘…features of social organisation such as networks, norms and social trust that facilitate co-ordination and co-operation for mutual benefit’ [11]. He argued that the presence of such ‘features’ within populations reflected the presence of social capital [12]. He further hypothesized that communities deemed rich in social capital also consisted of individuals with better health [13].

Though Putnam and others view social capital as a contextual phenomenon [14], any difficulties surrounding its measurement are often overcome by employing individual-level proxies, such as generalized trust and social participation [12,13]. To capture any contextual effect of social capital, researchers may further aggregate such proxies to a context of interest, typically a community-, state- or county-level [5,15,16]. However, such ‘classic’ contexts are often chosen more out of convenience than as accurate representations of individuals’ day-to-day social interactions and networks. As social networks are an integral part of the definition of social capital [13,14], analysis of inappropriate contexts may fail to capture any social capital effects [17]. The intra-class correlation (ICC), an expression of variance often employed in multilevel analyses, succinctly highlights this point. The ICC quantifies the proportion of residual variation of an outcome that is attributable to a specific context. In multilevel social capital research investigating health outcomes, the ICC is typically 0–4% for classic geographic contexts, such as the neighbourhood or community (for recent examples see [5,15,16,18]).

It is not surprising to find researchers attempting to identify a more appropriate cluster with which to investigate social capital, for example ‘the workplace’ [19,20]. However, workplace studies can (by definition) only sample working adults, with results being less readily extrapolated to general societal contexts.

Another context of interest, recently investigated in social capital research, is the ‘household’ [17]. The study by Giordano et al. [17] was based upon the premise that maintenance and formation of trust in others, and the propensity to participate socially was “affected by the close social context of the family and the household in which a person lives”. This premise is supported in past literature; for example, in the early 1980s, researchers discussed how levels of dyadic or ‘particularized’ trust between members of the same households could influence levels of generalized or ‘interpersonal’ trust (now a common social capital proxy) [21,22]. The social scientist James Coleman later postulated that a stable household was an important environment within which to generate higher levels of particularized and generalized trust, with both trust variants being needed to facilitate actions between individuals or groups (i.e. without trust there was no social capital) [23]. Fukuyama [24] and Putnam [13] also discussed the relationship between levels of trust, the household and social capital; however, scarcity of data sampled entirely at the household level (as opposed to sampling one member per household [25,26]) means that social capital research has been unable to fully explore this context.

Of the only empirical social capital research paper sampled on entire households, Giordano et al. [17] estimated an ICC of 25% for households, i.e. one quarter of the total variation in individual health (the ICC) could be attributable to the household context (compared with 2% for neighbourhoods in the same study). They further showed that only high levels of household-level trust (as opposed to individual- or neighbourhood-level trust) were positively associated with health. However, two important limitations of Giordano et al.’s (2011) study remain: firstly, it was cross-sectional in design; secondly, despite use of an ecometric approach to create contextual social capital variables, certain bias remained [27], with results possibly being confounded by unmeasured factors, such as genes or the shared environment.

A PubMed search revealed only one previous study that attempted to address such bias in social capital research. Fujiwara and Kawachi used a twin-pair study to investigate the association between generalized trust, participation and health [28]. Though Fujiwara and Kawachi’s cross-sectional study ruled out personality and early child environment as possible confounders, it ‘…does not necessarily control for all life course and adult factors on which twins may differ’, nor can it tackle issues of reverse causality [29].

The aim of our longitudinal and multilevel study, therefore, was to investigate the validity of associations between two of the most common social capital proxies (generalized trust and social participation [9,12,13]) and self-rated health (SRH), whilst employing a family-based design, sampling adults who shared the same environment (household) over a six-year period. By doing so, we intended to address the limitations of the studies highlighted above, with a view to furthering social capital research.

## Methods

The British Household Panel Survey (BHPS) is a longitudinal survey of randomly selected private households conducted by the United Kingdom’s (UK) Economic and Social Research Centre. Since 1991, all individuals aged sixteen years or older within selected households have been interviewed annually with a view to identifying social and economic changes within the British population. The original cohort sample was randomly selected by using a two stage cluster design, full details of which can be found on-line [30]. All data derived for this study were sampled on entire households.

The raw data for this longitudinal multilevel study come from BHPS participants providing responses for the years 2003, 2005, 2007 and 2008 (N = 6982), who were clustered on households (N = 4031). Each household contained between one and six adult respondents (eighteen years of age or older), who remained within the same household over the six-year study period. Past literature has shown that prior changes in social capital precede changes in SRH [31]. Based on this premise, and in an attempt to address issues of reverse causality not addressed in the twin study by Fujiwara and Kawachi (2008) [29], we derived all independent variables from 2003, 2005 and 2007 data and our outcome (SRH) from 2008. Participation rates for Wave thirteen (2003) compared to the original 1991 sample was 70.2%.

The Economic and Social Research Centre fully adopted the Ethical Guidelines of the Social Research Association, which conform to those of the International Statistical Institute. Informed consent was obtained from all BHPS participants by the Research Centre, and strict confidentiality protocols were adhered to throughout data collection and processing procedures. The author responsible (GNG) for data and analyses in this study signed a separate confidentiality agreement with the Research Centre to ensure confidentiality protocols were maintained during subsequent analyses.

### Dependent variable

Our outcome variable is SRH, which has been repeatedly found to be a valid predictor of mortality and morbidity [32,33]. In 2008, individuals were asked: ‘Compared to people your own age, would you say that your health has, on the whole, been excellent, good, fair, poor or very poor?’ As is standard with this outcome, this five-point scale was recoded as a dichotomous variable with the labels ‘good’ (excellent, good) and ‘poor’ (fair, poor, very poor), the outcome of interest being ‘poor’ health.

### Independent variables

We used the two most commonly used social capital proxies in this study: generalized trust and social participation [9,12,13]. Generalized trust was assessed by asking people: ‘Would you say that most people can be trusted, or that you can't be too careful?’ This variable was dichotomized into ‘can trust others’ (0) and ‘can’t trust others’ (1), with the response ‘most people can be trusted’ being the reference group. The responses ‘you can’t be too careful’ and ‘it depends’ were combined and given the value 1.

Levels of social participation within the community were measured by asking the respondents questions about being *active* members of any of the following group or organizations: Political party, trade union, environmental group, parents’ / school association, tenants’ / residents’ group or neighbourhood watch, church organization, voluntary service group, pensioners group / organization, social club / working men’s club, sports club and the Women’s Institute. Only those who answered positively to any of these elements were judged to participate socially, with responses being dichotomized into ’active participation’ (0) and ‘no participation’ (1).

As the above social capital proxies are time-dependent (i.e. respondents’ answers can vary from year to year), we summed the dichotomous (1–0) responses from years 2003, 2005 and 2007 and re-categorized them to reflect potential changes over time. Taking ‘trust’ as an example, those who could trust in all three waves (scoring ‘0’ in total after summing the values for the three waves) were labelled ‘always trusts others’; those who couldn’t trust in any of the three waves (scoring ‘3’ in total) were labelled ‘never trusts others’; any respondent scoring ‘1 or 2’ in total (reflecting a change in trust levels over time) were labelled ‘intermittent trust’. We repeated this process for the social capital proxy ‘social participation’. We utilised these summed social capital variables in our individual-level longitudinal analysis (Model 1 below).

In order to implement our multilevel investigation (Model 2 below), we first aggregated the summed individual-level social capital variables (from Model 1) on the group-centred mean (average) value, maintaining grouping on household clusters. This step produced the ‘*mean household value*’, a comparison *between* different households. We employed this continuous aggregate measure in our traditional (contextual) multilevel model.

**Figure 1:**
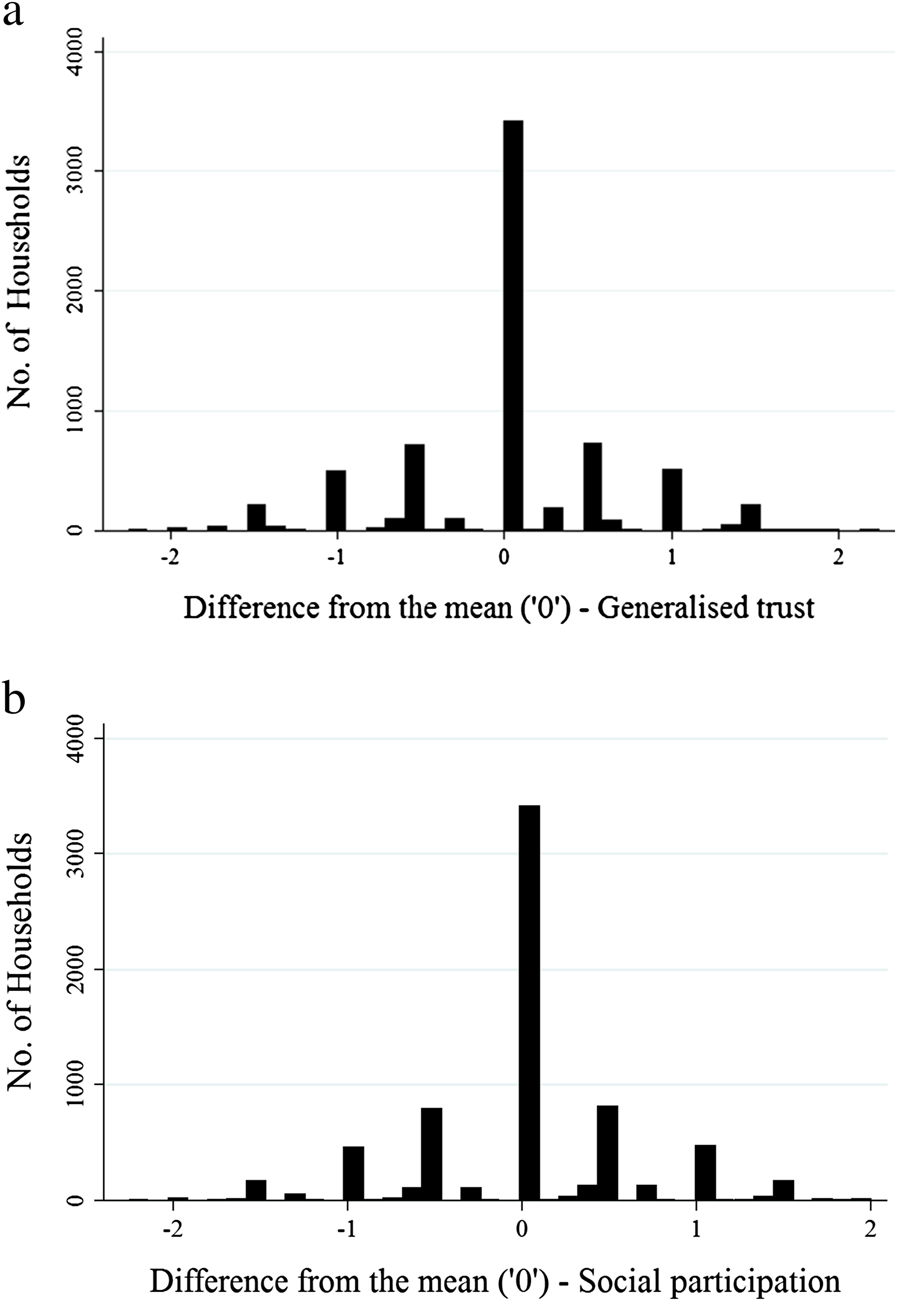
Distribution of within-household differences of social capital values around the mean (zero): (a) Trust (b) Participation.

Our family-based design further required us to identify those individuals who, despite sharing the same household for six years, had different social capital values. We created a new (compositional) social capital value by subtracting the minimum from the maximum group-centred mean aggregated social capital value for each household. Households that contained only one individual, or individuals with *identical* social capital values scored ‘zero’; all others would contribute to the ‘*difference from the mean*’ score, a compositional social capital measurement now *not* confounded by shared environment–see Figure 1a and b for the distributions of these social capital variables around the ‘mean’.

Highest achieved education, employment status and household income were included as measures of socio-economic status (SES). Household income was weighted according to size by summing the income of all household members and dividing this sum by the square root of the household size [34]. Income levels were expressions of total income, net of taxation.

As education can increase over time, the highest education level achieved was taken from 2007 data and categorized as ‘Undergraduate or higher’ , ‘Year 13’ , ‘Year 11’ and ‘No formal qualifications’. Employment status was categorized as ‘Employed’ , ‘Retired’ , ‘Fulltime student’ or ‘Unemployed’. Smoking status was categorized as ‘smoker’ and ‘non-smoker’ according to respondents’ answers to the question ‘Do you smoke cigarettes?’ As with our social capital proxies, smoking and employment status responses were summed across the three waves (2003, 2005 and 2007) to capture any change over time.

The other variables considered in this study were age and gender, with age being stratified into quintiles for all analyses (see tables).

### Statistical methods

For our individual-level model (Model 1), we ran all independent variables simultaneously against our outcome (poor SRH) in a multiple logistic regression analysis using Generalized Estimating Equations (GEE) [35], with an exchangeable working correlation structure, employing the ‘sandwich’ covariance estimator [36]. For our multilevel analysis (Model 2), we chose GEE over conventional multilevel modelling, as the assumption that residual variance was normally distributed did not hold for our data (possibly due to households containing only between 1–6 individuals [37]). Our two-level GEE model controlled for correlation at the household level, but provided no variance estimates.

Model 1 (Table 1) represents an individual-level longitudinal investigation into the association between ‘summed’ social capital variables and SRH.

**Table 1:** Odds ratios (OR) with 95% confidence intervals (95% CI) of poor self-rated health in 2008 according to individual-level multiple regression analysis of explanatory variables derived from 2003–07 (N = 6900)

**Explanatory variables**		**Poor self-rated health in 2008**
		**ORs (95% CI)**
Generalized trust	Always trusts others	1.0
	Intermittent trust	1.42 (1.22-1.67)
	Never trusts others	1.87 (1.59-2.19)
Social participation: Membership of local voluntary groups	Always active member	1.0
	Intermittent member	1.11 (0.96-1.28)
	Never active member	1.12 (0.96-1.31)
Age (years)	16–34	1.0
	35–44	1.37 (1.14-1.65)
	45–54	1.77 (1.47-2.13)
	55–64	1.37 (1.13-1.67)
	65 +	1.41 (1.12-1.79)
Gender	Male	1.0
	Female	0.76 (0.67-0.85)
Household income -size weighted	Per £1000 increase	1.00 ( 1.00-1.00)
Employment status	Employed	1.0
	Full time student	1.16 (0.97-1.39)
	Retired	1.85 (1.53-2.25)
	Unemployed	3.07 (2.55-3.69)
Highest education achieved	Undergraduate or higher	1.0
	Year 13	1.13 (0.94-1.35)
	Year 11	1.03 (0.87-1.22)
	No formal qualifications	1.60 (1.33-1.92)
Smoking status	Never a smoker	1.0
	Intermittent smoker	1.49 (1.24-1.80)
	Always a smoker	1.49 (1.29-1.72)

Source: The British Household Panel Survey, Waves M, O, Q & R (2003, 05, 07 & 08).

Reference group = 1.0.

Model 2 (Table 2) allows comparison of a traditional (contextual) multilevel design vs. our family-based (compositional) design. We use the ‘mean’ value to investigate *between* household differences (traditional (or contextual) multilevel modelling), and the ‘difference from the mean’ value to investigate *within-* household differences (an individual-level social capital measure (a compositional effect) now not confounded by shared environmental factors- our family-based design). All analyses were conducted within the statistical software package STATA 11.2 [38].

**Table 2:** **Odds ratios (OR) with 95% confidence intervals (95% CI) of having poor self-rated health in 2008: according to two-level multiple regression analysis using explanatory variables derived from 2003–07, simultaneously adjusting for *between *(contextual) and *within *(Family-based design–‘compositional’) household social capital measures (*N*** **= 6900)**

**Explanatory variables**		**Poor self-rated health in 2008**
		**ORs (95% CI)**
Generalized trust–*between* HHs	Mean value Traditional (contextual) ML design	1.29 (1.21-1.37)
Generalized trust–*within* HHs	Difference from the Mean Family-based design (compositional)	1.11 (1.02-1.20)
Social participation–*between* HHs	Mean value Traditional (contextual) ML design	1.07 (0.97-1.18)
Social participation–*within* HHs	Difference from the Mean Family-based design (compositional)	0.97 (0.89-1.06)
Age (years)	16–34	1.0
	35–44	1.39 (1.15-1.67)
	45–54	1.80 (1.49-2.17)
	55–64	1.41 (1.16-1.72)
	65 +	1.48 (1.16-1.88)
Gender	Male	1.0
	Female	0.76 (0.68-0.86)
Household income–size weighted	Per £1000 increase	0.99 (0.99-1.00)
Employment status	Employed	1.0
	Full time student	1.16 (0.97-1.40)
	Retired	1.84 (1.52-2.24)
	Unemployed	3.02 (2.51-3.64)
Education achieved	Undergraduate or higher	1.0
	Year 13	1.11 (0.93-1.32)
	Year 11	1.00 (0.84-1.18)
	No formal qualifications	1.52 (1.26-1.83)
Smoking status	Never a smoker	1.0
	Intermittent smoker	1.47 (1.22-1.76)
	Always a smoker	1.46 (1.26-1.69)

Source: The British Household Panel Survey, Waves M, O, Q & R (2003, 05, 07 & 08).

Reference group = 1.0.

HH = Household.

ML = Multilevel.

## Results

Table 3 shows frequencies and total percentages of all independent variables, stratified by SRH in 2008. Table 4 shows the number of respondents per household.

**Table 3:** Frequencies of all considered variables expressed as integers and percentages, stratified by self-rated health

		**Self-rated health**		
**Explanatory variables**		**Good**	**Poor**	**Total (N**_**T**_**)**
Generalized trust	Always trusts others	1380	330	1710
		*28.2%*	*15.8%*	*24.5%*
	Intermittent trust	1766	711	2477
		*36.1%*	*34.1%*	*35.5%*
	Never trusts others	1750	1045	2795
		*35.7%*	*50.1%*	*40.0%*
Total		4896	2086	6982
		*100.0%*	*100.0%*	*100.0%*
Social Participation: Active member of local groups, organisations or group leisure activities	Active participation	1341	470	1811
		*27.4%*	*22.5%*	*25.9%*
	Intermittent participation	2039	840	2879
		*41.6%*	*40.3%*	*41.2%*
	Zero participation	1516	776	2292
		*21.7%*	*11.1%*	*32.8%*
Total		4896	2086	6982
		*100.0%*	*100.0%*	*100.0%*
Gender	Male	2221	942	3163
		*45.4%*	*45.3%*	*45.3%*
	Female	2675	1144	3819
		*54.6%*	*54.8%*	*54.7%*
Total		4896	2086	6982
		*100.0%*	*100.0%*	*100.0%*
Age	16–34	1006	317	1323
		*20.5%*	*15.2%*	*18.9%*
	35–44	1190	391	1581
		*24.3%*	*18.7%*	*22.6%*
	45–54	1031	445	1476
		*21.1%*	*21.3%*	*21.1%*
	55–64	873	412	1285
		*17.8%*	*19.8%*	*18.4%*
	65+	796	521	1317
		*16.3%*	*25.0%*	*18.9%*
Total		4896	2086	6982
		*100.0%*	*100.0%*	*100.0%*
Employment status	Employed	3071	884	3955
		*62.7%*	*42.4%*	*56.6%*
	Full time student	630	221	851
		*12.9%*	*10.6%*	*12.2%*
	Retired	849	556	1405
		*17.3%*	*26.7%*	*20.1%*
	Unemployed	346	425	771
		*7.1%*	*20.4%*	*11.0%*
Total		4896	2086	6982
		*100.0%*	*100.0%*	*100.0%*
Highest education achieved ^a^	Undergraduate or higher	1304	327	1631
		*26.9%*	*15.9%*	*23.6%*
	Year 13	1032	364	1396
		*21.3%*	*17.7%*	*20.2%*
	Year 12	1583	594	2117
		*32.7%*	*28.8%*	*31.6%*
	No formal qualifications	921	775	1696
		*19.0%*	*37.6%*	*24.6%*
Total		4840	2060	6900
		*100.0%*	*100.0%*	*100.0%*
Smoking status	Never smokes	3733	1374	5107
		*76.2%*	*65.9%*	*73.1%*
	Intermittent smoker	432	233	665
		*8.8%*	*11.2%*	*9.5%*
	Always smokes	731	479	1210
		*14.9%*	*23.0%*	*17.3%*
Total		4896	2086	6982
		*100.0%*	*100.0%*	*100.%*
Household income (annual, size-weighted)	< £ 30,818	110	646	1746
		*22.5%*	*31.0%*	*25.0%*
	£30,819–£54,107	1125	620	1745
		*23.0%*	*29.7%*	*25.0%*
	£54,108–£86,659	1225	490	1745
		*25.6%*	*23.5%*	*25.0%*
	£86,660 +	1416	330	1746
		*28.9%*	*15.8%*	*25.0%*
Total		4896	2086	6982
		*100.0%*	*100.0%*	*100.0%*

Source: The British Household Panel Survey Waves M, O, Q & R (2003, 05, 07 & 08).

^a^ Missing N = 82.

**Table 4:** The frequency of individuals per household (4031)

		**Number of households**
**Number of individuals per household**	1	1378
	2	2385
	3	241
	4	25
	5	1
	6	1
	Total	4031

Source: The British Household Panel Survey, Waves M, O, Q & R (2003, 05, 07 & 08).

Table 5 is a ‘2 × 2’ table showing differences in generalized trust and self-rated health within households (*N*_household_ = 4031); Table 6 shows the differences in social participation and SRH health within households.

**Table 5:** A 2 × 2 table showing differences in generalized trust and self-rated health within households (N = 4031)

	**No difference in levels of trust**	**Different levels of trust**	***Total***
No difference in SRH	2106	966	3072
	*52.2%*	*24.0%*	*76.2%*
Difference in SRH	364	595	959
	*9.0%*	*14.8%*	*23.8%*
*Total*	2470	1561	4031
	*61.3%*	*38.7%*	*100.0%*

Source: The British Household Panel Survey Waves M, O, Q & R.

**Table 6:** A 2 × 2 table showing the differences in exposure of social participation and self-rated health at the household level (N = 4031)

	**No difference in exposure of participation**	**Difference in exposure of participation**	***Total***
No difference in exposure of SRH	2080	384	2464
	*51.6%*	*9.5%*	*61.1%*
Difference in exposure of SRH	992	575	1567
	*24.6%*	*14.3%*	*38.9%*
*Total*	3072	959	4031
	*76.2%*	*23.8%*	*100.0%*

Source: The British Household Panel Survey Waves M, O, Q & R.

### Model 1

#### Individual-level longitudinal regression analysis

As shown in Table 1, the odds ratios (ORs) for the association between social capital (trust and participation) and poor SRH increased as presence of social capital diminished (never trusts others: OR = 1.87 (95% confidence interval 1.59-2.19); never participates: OR = 1.12 (0.96-1.31)). There were also associations between lack of formal qualifications (OR = 1.60 (1.33-1.92)), being unemployed (OR = 3.07 (2.55-3.69)), smoking (OR = 1.49 (1.29-1.72)) and poor SRH. Being female seems to protect against poor SRH (OR = 0.76 (0.67-0.85)).

### Model 2

#### Traditional (contextual) multilevel vs. Family-based design (compositional) regression analysis

As shown in Table 2, there are now two OR per social capital proxy. The ‘*mean’* value represents the association between social capital and poor SRH when comparing *different* households with each other (this ‘between’ context comparison is most often seen in traditional multilevel designs), while the *‘difference from the household mean’* value reveals the association between trust, social participation and poor SRH when comparing individuals from *within the same* household (our family-based design). After adjustment for shared environment (the same household over six years), our results show that the association between social capital and SRH was heavily attenuated (generalized trust–*within* households: OR = 1.11 (1.02-1.20); social participation–*within* households: OR = 0.97 (0.89-1.06)).

Being a smoker (OR = 1.46 (1.26-1.69)), having no formal qualifications (OR = 1.52 (1.26-1.83)) and being unemployed (OR = 3.02 (2.51-3.64)) maintain their associations with poor SRH. Household income and gender appear to protect against poor SRH (OR = 0.99 (0.99-1.00) and 0.76 (0.68-0.86), respectively).

## Discussion

The aim of our study was to test the validity of the association between two commonly used social capital proxies (generalized trust and social participation) and SRH using longitudinal data sampled on entire households. The association between SRH and social participation was not significant in ‘traditional’ or ‘family-based’ models (see Table 2). The association between trust and SRH remained, but was attenuated (OR = 1.11 (1.02-1.20)). These results mirror past research investigating the temporal relationships between prior social capital levels and future SRH status, showing that only prior changes in trust are associated with future changes in SRH [31].

Trust and social participation have been considered valid social capital proxies for nearly two decades [12,13], and have been extensively used to demonstrate the independent influence of social capital on health outcomes. Past social capital literature suggests that the association between generalized trust and health is the more robust [9], resulting in trust being labelled an independent predictor of health [31,39].

Results from Model 1 (showing trust and, to a lesser extent participation, remaining positivity associated with SRH–see Table 1) mirror past studies, thus validating our data for readers. Model 2 further allows readers to see how ‘traditional’ (*between* household) multilevel results are attenuated after adjustment for the shared household environment.

To clarify, the *‘mean’* household value for trust in our study (OR = 1.29 (1.21-1.37)) provides a comparison *between different households* from across the UK (see Table 2). We concur with Carlin et al. [35] that this value holds little relevance regarding our aim to validate the association between social capital and SRH because unmeasured confounders, e.g. the shared environment, have yet to be adjusted for. Conversely, the *‘difference from the household mean’* value provides a direct comparison of individuals from *within the same* household, and takes into account unmeasured factors such as the shared environment and positive assortative mating [40,41]. A *within* context analysis, therefore, produces more valid measures of association than a *between* context analysis, the latter being confounded by unmeasured factors not controllable by traditional multiple regression modelling.

The *‘difference from the household mean’* measure of trust in Table 2, though derived from aggregation, is an individual-level social capital measure (a compositional effect) now not confounded by shared environmental factors. The corresponding OR (1.11 (1.02-1.20)), though heavily attenuated compared with our individual-level trust variable in Table 1 (1.87 (1.59-2.19)), remained significant. This result implies that generalized trust is still an important predictor of individual health [39], even after adjusting for the shared environment over time. However, one should consider the size of the effect of trust on health in comparison to other well-known individual health determinants (for example, from our study–‘unemployed’ (OR = 3.02 (2.51-3.64)).

### Strengths and limitations

A major strength of our study is that it is longitudinal and multilevel. Data are sampled on entire households, allowing the implementation of our family-based design, which adjusts for the shared household environment over time. That all respondents remain within the same household over the six-year study period implies that *‘difference from the household mean’* values provide a valid and reliable comparison of individuals *within* the same household, who have differences in social capital and SRH (see Tables 5 and 6). By including individual-level covariates, such as multiple SES proxies and other confounders, we ensured that well-known health determinants were also included in our analyses. All respondents sampled in our data provided face-to-face interviews rather than responses to postal questionnaires, across the six-year time-frame.

A major limitation of this study is that the BHPS sample was originally selected to reflect the UK population as a whole, and deliberately avoided oversampling of smaller communities. Only 70.2% of the original cohort sample selected in 1991 was still available by 2003, introducing further selection bias into this study. Though SRH is a subjective measure, it is still considered a valid and reliable predictor of mortality and morbidity [32,33]. Though we have stated that our design only considers the shared household environment, descriptive analysis revealed that 9.8% of our sample population (N = 682) were, in fact, directly related (i.e. live with brother, sister or parent). However, we chose to maintain clustering and subsequent analyses solely at the ‘household’ level due to issues of statistical power.

Another limitation is that we were unable to simultaneously investigate the neighbourhood context and the household in this study. This meant that we were unable to test the theory that the household, rather than being a confounder, may be a pathway through which a higher-level context influences health. However, as households were nested in communities, we assume that individuals sharing the same household were also similarly influenced by shared neighbourhood characteristics (e.g. high crime rates). Therefore, any differences *within* the same household (our ‘*difference from the mean value*’) will also take into consideration any higher contextual (i.e. neighbourhood) effects on social capital.

## Conclusions

The results of this study suggest that past associations between social capital (specifically trust and participation) and SRH may have been confounded by shared environmental factors not previously considered by researchers. In this study, the association between participation and SRH is fully attenuated; however, that trust remains associated with SRH (even after adjustment for residual confounding) adds to existing empirical evidence that generalized trust is an independent predictor of health [28,31,39,42].

## Competing interests

The authors declare that they have no competing interests.

## Authors’ contributions

GNG acquired the data. JM and HC conceived and created the study design. HC and GNG managed the datasets and performed the statistical analyses. All authors contributed to the drafting and revision of the manuscript. All authors read and approved the final manuscript.

## Pre-publication history

The pre-publication history for this paper can be accessed here:

http://www.biomedcentral.com/1471-2458/13/665/prepub
